# Utility of *BRAF *V600E mutation detection in cytologically indeterminate thyroid nodules

**DOI:** 10.1186/1742-6413-3-10

**Published:** 2006-04-10

**Authors:** Leslie R Rowe, Brandon G Bentz, Joel S Bentz

**Affiliations:** 1 Institute for Clinical and Experimental Pathology, Associated Regional and University Pathologists (ARUP) Laboratories, Salt Lake City, UT, USA; 2 Division of Otolaryngology, Department of Surgery, University of Utah, Salt Lake City, UT, USA; 3 Department of Pathology, University of Utah, Salt Lake City, UT, USA

## Abstract

**Background:**

Fine needle aspiration (FNA) is widely utilized for evaluation of patients with thyroid nodules. However, approximately 30% are indeterminate for malignancy. Recently, a mutation in the *BRAF *gene has been reported to be the most common genetic event in papillary thyroid carcinoma (PTC). In this retrospective study, we assessed the utility of *BRAF *V600E mutation detection for refining indeterminate preoperative cytologic diagnoses in patients with PTC.

**Methods:**

Archival indeterminate thyroid FNAs and corresponding formalin-fixed, paraffin-embedded (FFPE) surgical samples with PTC were identified in our patient files. DNA extracted from slide scape lysates and 5 μm FFPE sections were evaluated for the *BRAF *V600E mutation using LightCycler PCR and fluorescent melting curve analysis (LCPCR). Amplification products that showed deviation from the wild-type genomic DNA melting peak, discordant FNA and FFPE matched pairs, and all benign control samples, underwent direct DNA sequencing.

**Results:**

A total of 19 indeterminate thyroid FNAs demonstrating PTC on FFPE surgical samples were included in the study. Using *BRAF *mutation analysis, the preoperative diagnosis of PTC was confirmed in 3/19 (15.8%) FNA samples that could not be conclusively diagnosed on cytology alone. However, 9/19 (47.4%) FFPE tissue samples were positive for the V600E mutation. Of the discordant pairs, 5/6 FNAs contained less than 50% tumor cells.

**Conclusion:**

When used with indeterminate FNA samples, *BRAF *mutation analysis may be a useful adjunct technique for confirming the diagnosis of malignancy in an otherwise equivocal case. However, overall tumor cell content of some archival FNA smear slides is a limiting factor for mutation detection.

## Background

While the frequency of thyroid cancer in the general population is relatively low, thyroid nodules are a very common clinical problem, and palpable thyroid nodules can be identified in 4–7% of all adults in the United States [1]. The prevalence of malignancy, however, in a solitary thyroid nodule is only approximately 5% in normal adults [2-4]. Consequently, the primary clinical challenge is to sort out the vast majority of nodules that are benign, which can generally be followed with surveillance, from those requiring surgical intervention.

In 2003, approximately 75–80% of all thyroid cancers were papillary thyroid carcinoma (PTC) [5]. Among the most curable of cancers, PTC tends to remain localized in the thyroid gland, but in time it may metastasize to regional lymph nodes and, less commonly, to the lungs. At the time of initial assessment, most patients with PTC present with a painless, palpable, solitary thyroid nodule.

As early as the1930's, studies reported on the use of fine needle aspiration (FNA) cytology for the diagnosis of thyroid carcinoma [6,7]. However, as often as 30% of the time, FNA-based evaluation of solitary thyroid nodules displays limited ability to discriminate between benign and malignant lesions and an indeterminate cytologic diagnosis is rendered [8]. Although surgical intervention is generally recommended following an indeterminate finding on FNA cytology, malignancy within indeterminate thyroid nodules varies between 3–52% [9-16]. Consequently, planning optimal surgical management in patients with an uncertain preoperative diagnosis is challenging.

In view of the increasing number of thyroid nodules that require FNA evaluation, there is a clear need for the development of adjunctive diagnostic assays that would help refine indeterminate diagnoses on thyroid cytology. Recently, a single hotspot mutation at nucleotide 1799 of the *BRAF *gene has been identified as the most common genetic event in 29–83% of all cases of PTC [17-27]. This thymine (T) to adenine (A) transversion mutation results in the substitution of valine with glutamate (V600E) and converts *BRAF *into a dominant transforming protein that causes constitutive activation of the MAPK pathway, independent of RAS activation [28]. Additionally, this mutation appears to be fairly specific for PTC.

In early polymerase chain reaction (PCR) testing platforms, sample DNA or RNA was amplified first and then detected in a separate step, using a technique such as gel electrophoresis to assess the size and purity of the products. Recently developed instrumentation combines PCR amplification and target nucleic acid characterization in the same closed reaction vessel [29]. Using LightCycler PCR with fluorescent melting curve analysis (LCPCR), the difference in melting profiles between mismatched probe/target and perfectly matched probe/target can be used to characterize amplification products and indicate the presence of a mutation [30].

The primary objective of this study was to identify the *BRAF *V600E mutation in thyroid FNA samples using LCPCR for the purpose of refining indeterminate preoperative cytologic diagnoses in patients with PTC.

## Materials and methods

### Case and sample selection

**Figure 1:**
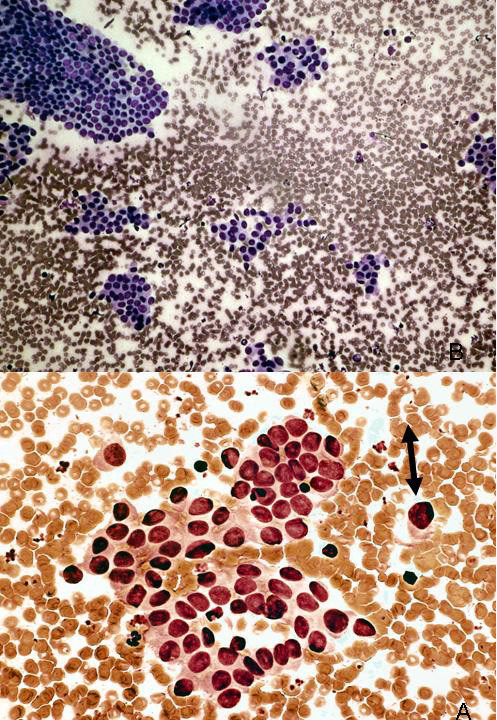
Example of indeterminate thyroid fine needle aspiration (FNA). Fine needle aspirate sample from 28 year-old female with thyroid nodule. Case was interpreted as "fragments of atypical epithelial cells in a background of reactive lymphocytes. A follicular neoplasm or papillary carcinoma cannot be excluded." The thyroidectomy specimen showed a classical papillary thyroid carcinoma in the corresponding lobe of the thyroid.

This retrospective study was approved by the University of Utah Institutional Review Board (#13005). Study samples were identified from a database established by the University of Utah Department of Surgery (University of Utah Institutional Review Board #11565) of all patients having undergone treatment for cancer of the thyroid gland at the University of Utah Hospitals and Clinics from 1994–2004. This dataset of patients was reviewed to identify only individuals with both an archival, preoperative FNA demonstrating cytologic findings indeterminate for malignancy and follow-up archival surgical pathology formalin-fixed, paraffin-embedded (FFPE) tissue demonstrating PTC. The "indeterminate" cytologic category encompassed those samples demonstrating hypercellularity suggestive of a follicular or papillary neoplasm and/or atypical cytomorphologic features suggestive of, but not diagnostic for, malignancy (Figure 1). The diagnostic correlation was restricted to cases in which the cytology was reported as indeterminate within a 6-month period preceding the histology report.

The archival cytologic slides were retrieved from the cytopathology laboratory slide archives. Only previously-coverslipped and Diff-Quik stained direct smear slides were pulled for DNA extraction and mutation analysis. A case was selected for study inclusion if: age greater than 18; indeterminate cytology report; cytology slide appeared to contain more than 15% cellularity; follow-up surgical pathology tissue block was available; and the use of a single archival cytology slide did not compromise the patient's permanent slide record. Once identified as available for mutation analysis, the patient's cytopathology report was reviewed in order to establish the final cytologic diagnosis and document the area of the thyroid having undergone FNA.

The surgical pathology tissue blocks were retrieved from the surgical pathology laboratory tissue block archives. A cytopathologist (JSB) reviewed the patient histopathology reports in order to select only the tissue block that corresponded to the nodule that underwent FNA. If the cytopathology report identified the FNA site as "thyroid-not otherwise specified, " the tissue block for analysis were selected from nodules demonstrating PTC.

Finally, we identified matched pairs of preoperative thyroid FNA and follow-up FFPE thyroid tissue samples demonstrating benign findings (e.g. nodular goiter, thyroiditis). This set of samples served as negative controls for the LCPCR assay.

### DNA isolation from FFPE tissue sections

Five 5 μm sections were cut from the FFPE tissues. One tissue section was hematoxylin and eosin (H/E) stained and coverslipped for review by a cytopathologist (JSB). Using a pen, the area of the tissue that contained the tumor was marked. The remaining unstained sections were deparaffinized by immersion in 3 changes of xylene for 5 minutes each. The tissue sections were then hydrated in a graded series of ethanol, followed by immersion in dH_2_O for 1 minute. The slides were allowed to air-dry completely. The unstained tissue section slide was then inverted over the H/E-stained slide and the area identified by the cytopathologist was marked on the underside of the unstained tissue section slide. Using the circled area of interest on the unstained tissue section slide as a guide, a scalpel blade was used to manually scrape the areas of the tissue containing the tumor cells of interest. Following manual microdissection, the scalpel blade was inserted into a clean microcentrifuge tube. A 25.0 μl aliquot of Proteinase K (3 mg/ml) digestion solution (50 mM Tris, 1 mM EDTA, pH8.0, 1% Tween 20) was pipetted onto the scraped area of the slide to pick up any remaining cells. The digestion solution was then pipetted from the slide and used to rinse the scalpel blade that was positioned inside the labeled microcentrifuge tube.

### DNA isolation from FNA samples

A cytopathologist (JSB) assigned each FNA smear slide a score on based on overall cellularity and atypical cell content. Overall cellularity scores were as follows:

1+: Unsatisfactory (≤ 1 or 2 clusters of epithelial cells); 2+: Scant (3–10 clusters of epithelial cells) 3+: Adequate (10–20 clusters of epithelial cells) 4+: Abundant (> 20 clusters of epithelial cells with most fields of cells touching). Atypical cell content scores were as follows: 1+: < 25% atypical cells; 2+: 25–50% atypical cells; 3+: 50–75% atypical cells; 4+: >75% atypical cells.

Using a pen, the area of the archival FNA slide containing area of atypical cells of interest was marked. A diamond-tipped pencil was then used to mark the underside of the slide indicated by the cytopathologist. Slide coverslips were detached in xylene, and the slides were hydrated in a graded series of alcohol, followed by soaking in distilled water for 2 minutes. The FNA slides were then hydrated in a graded series of ethanol, followed by immersion in dH_2_O for 1 minute. The slides were allowed to air-dry completely. Using the area of interest indicated by the diamond-tipped pencil marking on the FNA slide as a guide, slide scrape lysates (SLL) were prepared by using a single-edged razor blade to scrape the areas of the slide containing the atypical cells of interest. Following manual microdissection, a 50.0 μl aliquot of Proteinase K (3 mg/ml) digestion solution (50 mM Tris, 1 mM EDTA, pH 8.0, 1% Tween 20) was pipetted onto the scraped area of the slide to pick up any remaining cells. Using the same pipette tip, the digestion solution was then pipetted from the slide and used to rinse the scalpel blade that was positioned inside the labeled microcentrifuge tube.

### DNA extraction

The samples were incubated in the digestion solution at 55°C for 12–16 hours. Following centrifugation at 12,000 rpm for 5 minutes, the supernatant was transferred into a newly labeled microcentrifuge tube. The samples were then placed into a 95°C heat block for 10 minutes to inactivate the proteinase K. All FFPE tissue sample DNA was diluted to a working concentration of 50 ng/μl prior to amplification. A MicroSpin G25 (Amersham Bioscience, Sweden) sephadex column was routinely used for all FNA samples following DNA extraction. Fine needle aspiration sample DNA then underwent PCR without additional dilution. Following DNA extraction, all samples were stored at -20°C prior to analysis.

### Characterization of control material

To assess the sensitivity and specificity of the LCPCR method for detection of the *BRAF *V600E mutation, three cell lines were analyzed. A human PTC-derived cell line (NPA) was characterized for use as a positive control, while one follicular thyroid carcinoma (ROW-1), and one colorectal carcinoma (HCT116) cell lines were characterized for use as negative controls. All cell lines were grown to a concentration of 3 × 10^6 ^cells per ml, trypsinized, and transferred to a 1.5 ml microcentrifuge tube. Cell line DNA was isolated and purified using the QIAamp DNA Mini Kit (QIAGEN Inc., Valencia, CA). DNA concentration and purity was determined and all cell lines were diluted to a working concentration of 50 ng/μl and stored at 4°C.

### Polymerase chain reaction and fluorescent melting curve analysis

For this study, a pair of oligonucleotide primers were designed to amplify a 250 base-pair region of exon 15 in the *BRAF *gene:

forward: 5'CTCTTCATAATGCTTGCTCTGATAGG-3 and

reverse: 5'TAGTAACTCAGCAGCATCTCAGG-3' (Integrated DNA Technologies, Inc, Coralville, IA).

Two fluorescent hybridization probes were designed to detect the *BRAF *V600E mutation: ^23 ^sensor: 5'-AGCTACAGTGAAATCTCGATGGAG-Fluoroscein-3' and

anchor: 5'-LCRed640-GGTCCCATCAGTTTGAACAGTTGTCTGGA-Phosphate-3'

with the sensor probe spanning nucleotide position 1799 (Idaho Technologies, Salt Lake City, UT). Amplification was performed in glass capillaries using 50 ng of tissue or FNA (range 12.0 to 110.0 ng) sample DNA in a 10 μl volume containing 1 ul of 10× LightCycler DNA Master Hybridization Probes (Roche Molecular Biochemicals, Mannheim), 0.8 μl of 25 mM MgCl_2_, 1 μl (5 μM) forward and reverse primer, and 1 μl (2 μM) anchor and sensor hybridization probe. The reaction mixture underwent 45 cycles of rapid PCR. Post amplification fluorescent melting curve analysis was performed by gradual heating of the samples at a rate of 0.1°C per second from 45°C to 95°C. Fluorescent melting peaks were determined by plotting of the negative derivative of fluorescence (F) with respect to temperature (T), or -dF/dT.

### Limit of detection experiment

A limit of detection experiment was first conducted to determine the percent of tumor with normal cell contamination in which abnormal melts were detectable by LCPCR. The NPA cell line was diluted with human genomic wild-type (WT) DNA to 99% tumor, 95% tumor, 90% tumor, 75% tumor, 50% tumor, 25% tumor, 10% tumor, and 5% tumor. In addition, 100% NPA and 100% WT samples were tested. Each dilution was run in duplicate.

### DNA sequencing analysis

All PCR products that showed deviation from the WT genomic DNA melting peak, as well as any discordant FNA and FFPE matched pairs, and benign control samples were confirmed by direct sequencing of exon 15. Ten ul of amplified sample plus 1.0 ul of Biotracker 6× tracking dye (Bioventures, San Francisco) was loaded into the sample wells of a 2% agarose DNA gel and electrophoresed at 70 volts. To isolate the DNA from the agarose gel, the desired ethidium-stained band was viewed with a UV transilluminator and excised using a razor blade. Sample DNA was then extracted from the agarose gel using a nebulizer (Millipore, Bellirica, MA). Bidirectional DNA sequence data was generated for each sample using fluorescently labeled terminator sequencing chemistry and sequencing primers (5' primer and 3' primer). DNA sequencing data files from the purified sequencing reaction products were generated using an ABI Prism 3100 Genetic Analyzer (Applied Biosystems).

## Results

A total of 24 archival FNA (indeterminate n = 19; benign n = 5) and FFPE matched pair samples were evaluated for the *BRAF *V600E point mutation using LCPCR.

### Characterization of control material

A homozygous T→A mutation was identified in the *BRAF *gene (exon 15) at nucleotide 1799 in the NPA cell line, which is derived from PTC. The NPA cell line served as a positive control for *BRAF *V600E mutation analysis by LCPCR. To assess the specificity of the LCPCR method for detection of the *BRAF *V600E mutation, cell lines ROW-1 (follicular thyroid carcinoma) and HCT116 (colorectal carcinoma) underwent mutation analysis. Both ROW-1 and HCT115 cell lines showed a WT *BRAF *sequence. These findings were confirmed by direct DNA sequencing.

### LCPCR limit of detection

Results of the limit of detection experiments confirmed that the 1 base pair change in the *BRAF *mutation was detectable down to the level of 25% tumor when a homozygous mutant cell line was used as a control. Consequently, it was determined that the results of LCPCR for detection of the heterozygous *BRAF *V600E mutation in FNA or FFPE samples containing less than 50% tumor cells may not be accurate.

### Prevalence of *BRAF *V600E mutation in indeterminate thyroid fine needle aspirates

Using LCPCR and DNA sequencing, *BRAF *mutation analysis confirmed the preoperative diagnosis of PTC in 3/19 (15.8%) of the cases in the indeterminate group. Nine of 19 (47.4%) corresponding FFPE surgical samples collected from the same patients were positive for the *BRAF *mutation (Table 1).

**Table 1:** Clinicopathologic features and *BRAF *V600E mutation analysis results of 19 indeterminate thyroid FNA cases. Median patient age = 40 years.

Age	Sex	Cytologic Interpretation	Mutation Status FNA	Sequencing Result FNA	Surgical Pathology	Mutation Status Tissue	Sequencing Result Tissue
59	M	Highly atypical cells suspicious for follicular or papillary neoplasm	WT	WT	PTC; FV	MUT	MUT
34	F	Atypical follicular epithelium, cannot exclude follicular neoplasm	WT	WT	PTC	MUT	MUT
32	F	Atypical cells present suspicious for PTC	MUT	MUT	PTC	MUT	MUT
31	F	Atypical follicular cells; cellular pattern consistent with follicular neoplasm	MUT	MUT	PTC	MUT	MUT
54	M	Hurthle cell neoplasm with cystic degeneration	WT	WT	PTC; FV	MUT	MUT
13	F	Atypical cells suspicious for papillary or follicular lesion	WT		PTC	WT	
53	F	Atypical follicular cells consistent with follicular neoplasm	MUT	MUT	PTC	MUT	MUT
43	F	Suspicious but not diagnostic of PTC	WT		PTC	WT	
23	M	Highly atypical cells present suspicious for PTC	WT		PTC; FV	WT	
26	F	Follicular proliferation most consistent with a nodular goiter	WT	WT	PTC	MUT	MUT
37	M	Atypical folllicular epithelim, cannot exclude a follicular neoplasm	WT		PTC	WT	
26	M	Atypical cells suspicious for PTC	WT		PTC	WT	
55	M	Suspicious for a follicular neoplasm	WT		PTC	WT	
29	F	Atypical cells present suspicious for follicular or papillary neoplasm	WT		PTC	WT	
57	F	Atypical follicular cells in a background of lymphoid cells	WT	WT	PTC	MUT	MUT
54	F	Follicular neoplasm cannot be excluded	WT		PTC; FV	WT	
50	F	Follicular neoplasm cannot be excluded	WT		PTC; FV	WT	
28	F	Fragments of atypical epithelial cells; cannot exclude follicular neoplasm or PTC	WT	WT	PTC	MUT	MUT
64	F	Hyperplastic nodule vs. follicular neoplasm	WT		PTC	WT	

PTC = papillary thyroid carcinoma; FV = follicular variant; WT = wild-type *BRAF *V600E genotype; MUT = mutant *BRAF *V600E genotype (exon 15)

There was a 69% rate of concordance for *BRAF *mutation status between paired indeterminate FNA and malignant FFPE tissue specimens. Six FNA samples were negative for the *BRAF *V600E mutation, while the corresponding FFPE tissue samples were positive. Of these, 5/6 FNA samples contained less than 50% tumor cells. Conversely, an indeterminate FNA containing >75% atypical cells failed to demonstrate the mutation, while the resected tumor was positive for the mutation. The overall cellularity of this sample was scored at a 2+.

**Figure 2:**
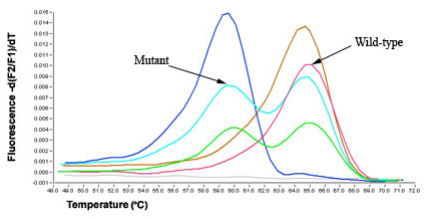
Melting curve analysis of *BRAF *mutations in thyroid FNA samples. Overlapping fluorescein-labeled oligonucleotide probes were used to scan extracted DNA for mutations in exon 15 of *BRAF*. Multiple probes complementary to the wild-type (WT) sequences were placed within the same reaction, and the different sites were identified by their specific probe/target duplex melting temperatures. The position of each probe/target melting temperature and the relative ratio of the melting peak areas determined WT profiles. After amplification in a LightCycler, the instrument begins a melting program where the reactions are cooled to anneal the probes and then slowly heated (0.1°C/s) while fluorescence is continuously monitored. Somatic mutations are identified by changes from a characteristic WT melting curve profile. When melting curves from non-mutated and mutated samples are compared, additional melting peaks or changes in peak-area ratios indicate a sequence alteration (nucleotide mismatch) under the probe. Melting curve analysis revealed that for the WT *BRAF *sequence (GTG) Tm was 65.34°C ± 0.37°C, the GTG→GAG mutation at nucleotide 1799, resulted in a shift to 60.23°C ± 0.53°C.

Melting curve analysis revealed that for the FNA samples, the WT sequence (GTG) Tm was 65.34°C ± 0.37°C, the GTG→GAG mutation at nucleotide 1799, resulted in a shift of Tm to 60.23°C ± 0.53°C (Figure 1). For the FFPE samples, the WT Tm was 64.92°C ± 0.35°C, while the mutant Tm was 60.11°C ± 0.46°C (Figure 2).

Bidirectional DNA sequencing revealed that all *BRAF *mutations were heterozygous and involved a T→A substitution at nucleotide 1799. The LCPCR assay demonstrated 100% concordance between melting curve and DNA sequence results. 0/5 benign thyroid FNA and matching FFPE samples were also found to be negative for the mutation.

## Discussion

As often as 30% of the time, FNA cytology displays limited ability to discriminate between benign and malignant thyroid lesions, and an indeterminate diagnosis is rendered [31]. Some clinicians feel that total thyroidectomy is appropriate for patients with an indeterminate FNA cytology result [9,10,32]. Proponents of this approach argue that it eliminates the probability of thyroid cancer recurrence [33-36]. Alternatively, if the suspect nodule is small, some clinicians opt to perform a hemi-thyroidectomy procedure following an indeterminate cytology result [37]. Considered the minimum extent of surgery for a thyroid nodule, this procedure reduces the risk of postoperative complications associated with total thyroidectomy, such as hypoparathyroidism and laryngeal nerve injury [38]. However, depending on patient/tumor risk stratification, postoperative confirmation of malignancy usually results in a second-stage completion thyroidectomy, which is associated with higher morbidity than initial total thyroidectomy [39]. Regardless of the choice of surgical procedure, the incidence of malignancy in patients with indeterminate cytology findings varies greatly. Consequently, a large percentage of these patients would benefit from a method that improves the diagnosis of preoperative thyroid aspirate material.

A number of molecular markers have been evaluated as possible adjunct tests for refining the diagnosis of PTC on FNA. However, the predictive value of these markers has been limited to date due to a lack of specificity or sensitivity [40]. Expressed by malignant thyrocytes, galectin 3 is a β-galactoside-binding protein that was initially believed to be a marker specific for PTC [41]. Further analysis has revealed, however, that identification of this protein may be less reliable in conditions involving lymphocytic infiltration, such as Hashimoto's thyroiditis [42]. HBME-1 is a monoclonal antibody that recognizes an epitope expressed in malignant mesothelioma and other adenocarcinomas, as well as PTC and follicular thyroid tumors [43,44]. Although benign thyroid lesions do not express immunoreactivity for HBME-1 [44], positive staining has been found in malignant thyroid tumors besides those of papillary differentiation [45]. Telomerase is a specialized reverse transcriptase enzyme that maintains chromosome ends. Detection of telomerase expression by reverse-transcriptase PCR originally showed promising sensitivity and specificity for PTC diagnosis [46], but telomerase repeat amplification (TRAP) has identified high expression of this molecule in FNA specimens from benign nodules [47]. Up to 95% of PTC demonstrate strong immunostaining with cytokeratin 19 (CK19) [48]. However, CK19 immunoreactivity is not specific for PTC, as positive immunoreactivity has been identified in benign follicular adenomas [49]. Aberrant expression of the RET proto-oncogene results from chromosomal rearrangements in which the tyrosine kinase domain of RET is fused to the 5'-terminal region of an unrelated gene, leading to the generation of fusion proteins known as ret rearrangements in PTC (RET/PTC). Although RET/PTC rearrangements have been identified in a large percentage of PTC in individuals exposed to external radiation [50], a relatively high frequency of RET/PTC rearrangements have also been found in benign nodular thyroid diseases of patients exposed to nuclear fallout and in benign conditions such as trabecular adenomas and Hashimoto's thyroiditis [51].

The mutation at V600E in the *BRAF *kinase gene appears to be an attractive molecular marker for thyroid cancer diagnosis as it has been found to be the most common genetic event in PTC, while being highly specific for this tumor (Table 2). The goal of this study was to identify the *BRAF *V600E mutation in thyroid FNA samples in an attempt to determine if *BRAF *mutation analysis can serve as a useful adjunct technique in indeterminate cytologic diagnoses. The use of LCPCR was chosen for SNP detection because both gene amplification and allele analysis could be performed in a homogeneous, closed-tube system on the same instrument. Increased specificity for mutation detection is realized due to the hybridization of two independent probes and the fact that the probe melting temperature is sequence specific. Of the cases evaluated in this study, which included 24 matched pairs of FNA and FFPE surgical tissues, there were no false positive *BRAF *mutation results by LCPCR and LCPCR demonstrated 100% concordance with DNA sequencing results.

**Table 2:** *BRAF *V600E mutation prevalence in various thyroid neoplasms.

**Series**	**FA**	**FTC**	**MTC**	**PTC (%)**
Cohen et al. J Natl Cancer Inst 2003	0/20	0/13	0/3	24/35 (69)
Kimura et al. Cancer Res 2003	0/14	0/10		28/78 (36)
Fukushima et al. Oncogene 2003		0/8	0/9	40/76 (53)
Soares et al. Oncogene 2003	0/51	0/18		23/50 (46)
Namba et al. J Clin Endocrinol Metab 2003	0/20	0/11		49/170 (29)
Nikiforova et al. J Clin Endocrinol Metab 2003	0/46	0/32	0/13	45/119 (38)
Xu et al. Cancer Res 2003	0/18			21/56 (38)
Kim et al. Yonsei Med J 2004				58/70 (83)
Trovisco et al. J Pathol 2004				28/53 (53)
Xing et al. J Clin Endocrinol Metab 2004	0/43	0/14	0/14	17/38 (45)
Salvatore, G et al. J Clin Endocrinol Metab 2004	0/19			26/69 (38)
Total	0/231	0/106	0/37	359/814

FA = follicular adenoma; FTC = follicular thyroid carcinoma; MTC = medullary thyroid carcinoma; PTC = papillary thyroid carcinoma

Previous investigators have also reported on use of the LCPCR method for detecting the V600E activating point mutation in the *BRAF *gene. In contrast to the present study, which analyzed archival FNA and FFPE surgical material for mutation detection, these groups utilized primarily cell lines or a combination of cell lines and FFPE surgical material. Nikiforova et al. evaluated thyroid tumors and anaplastic carcinoma cell lines to demonstrate that *BRAF *mutations, which in thyroid tumors were originally thought to be restricted to papillary carcinomas, also occur in poorly differentiated and anaplastic carcinomas [23]. Among 259 thyroid tumor samples screened, this group demonstrated a 100% correlation in *BRAF *V600E detection rate between LCPCR and single strand conformational polymorphism. This finding is in agreement with the results of the present study, in which LCPCR assay demonstrated 100% concordance between melting curve and DNA sequencing results. In contrast to the present study, however, Nikiforova used laser capture microdissection (LCM) to obtain DNA either from a small focus of papillary microcarcinoma or to study well-differentiated and poorly differentiated or anaplastic areas within the same tumor. Ikenoue et al. analyzed 12 colon and 9 gastric cancer cell lines by LCPCR for presence of the *BRAF *V600E mutation [52]. Using a mixture of standard DNA, Ikenoue determined that as little as 10% V600E mutant DNA could be identified in a background of WT DNA. In the present study, results of the limit of detection experiments confirmed that the 1 base pair change in the *BRAF *mutation was detectable down to the level of 25% tumor when a homozygous mutant cell line was used as a control. Consequently, it was determined that the results of LCPCR for detection of the heterozygous *BRAF *V600E mutation in FNA or FFPE samples containing less than 50% tumor cells may not be accurate. It is s possible that the level of sensitivity might be increased through the use of a technique such as LCM of the archival FNA slide material. As demonstrated by Nikiforova, et al, using LCM can significantly enhance the sensitivity for identifying mutant DNA in the presence of WT DNA, as the captured sample contains almost exclusively tumor cells.

In the present study, the preoperative diagnosis of PTC was confirmed in 3/19 (15.8%) indeterminate FNA samples that could not be conclusively diagnosed by cytology alone. This finding is consistent with reported mutation prevalence rates in indeterminate thyroid FNA cases (Table 3) [27,53,54]. However, 9/19 (47.4%) corresponding FFPE surgical samples collected from the same patients were positive for the mutation, for a 69% rate of concordance between the sample types. Of the 6 discordant FNA cases in this study, 5/6 (83%) contained <50% atypical cells. Because tumor DNA from FNA samples is invariably contaminated with the WT allele of the gene in question, the somatically mutated allele can be difficult to distinguish. This results in reduced sensitivity for identifying mutant DNA in the presence of WT DNA. Results of the limit of detection experiments confirmed that the one base pair change in the *BRAF *mutation was detectable by LCPCR down to the level of 25% tumor when a homozygous mutant cell line was used as a control. Because the *BRAF *V600E mutation in PTC is heterozygous, a detection limit of 50% atypical cells was established for the LCPCR assay, below which the results of LCPCR might not be accurate. Because LCPCR and DNA sequencing results were in 100% agreement for all samples, it is likely that the percentage of *BRAF *mutant DNA in the discordant FNA samples was below the limit of detection for both methods.

**Table 3:** *BRAF *V600E mutation prevalence rates in indeterminate thyroid FNA cases

**Series**	**Number of indeterminate thyroid FNA's evaluated**	**Number (%) with *BRAF *V600E mutation**
Salvatore, *et al. *2004	15	4 (27)
Cohen, *et al. *2004	32	5 (16)
Xing, *et al. *2004	25	2 (8.3)
Total	72	11 (15.3)

Other investigators have also experienced varying degrees of concordance for *BRAF *mutation status between matched FNA and FFPE samples. Cohen et al. noted discordant results in 3/49 matched pair samples for a 94% rate of concordance between the sample types [53]. Of the three discordant FNA samples in Cohen's study, the mutation was not detected in 2 FNAs while the resected tumors harbored the mutation. In these 2 cases, the FNA material was found to be sparsely cellular. These previous findings, combined with those of the present study, confirm that overall tumor cell content of the FNA sample is critical for mutation detection, whether by LCPCR or another method.

If the results of the limit of detection experiments in the present study were used to select samples for mutation analysis, thereby excluding from analysis any FNA sample that contained <50% atypical cells, 2/8 indeterminate FNA samples would have been found to harbor the *BRAF *mutation. The resulting *BRAF *positivity rate in the indeterminate FNA samples would, therefore, be 25% rather than 15.8%. In actual clinical practice, the pathologist would control pre-analytic probability by carefully selecting which indeterminate thyroid FNAs would be referred for mutation analysis, based upon the cellular composition of the smears and/or the needle rinse pellet.

It is also possible that the discordant aspirate material in the present study contained only the *BRAF *WT genotype, and that the mutant DNA identified in the resected tumor samples was the result of an early tumorigenic event. However, this is an unlikely scenario, as inclusion criteria allowed cases to be selected for mutation analysis only if the cytology was reported as abnormal within a six-month period preceding the histology report. This restriction was instituted in an attempt to reduce mutation status discordance between the sample types. Five of the six discordant FNA cases were collected within one month prior to the FFPE surgical tissue.

Because PTC is frequently multifocal, there has been speculation regarding whether noncontiguous tumor foci arise from intraglandular metastases from a single primary tumor or originate as unrelated clones derived from independent tumors. A recent study by Shattuck et al. [55] used PCR to evaluate the patterns of X chromosome inactivation of multiple distinct foci of PTC from 17 women. Discordant patterns indicative of independent origins were identified in tumors from 5 patients, leading to the conclusion that individual tumor foci in patients with multifocal PTC often arise as independent tumors. This finding could explain why an indeterminate FNA containing >75% atypical cells in the present study failed to demonstrate the mutation, while the resected tumor was positive for the mutation. The possibility exists that the tumor identified in the resected tissue, and in the nodule sampled by FNA, did not share the same clonal origin.

While the hotspot mutation at V600E is the most common genetic event in PTC, recent studies have demonstrated that up to 9% of follicular variant of PTC (FVPTC) cases demonstrate a mutation in codon 601 of the *BRAF *gene, resulting in the substitution of lysine with glutamate (K601E) [25,56]. Although five of the indeterminate thyroid FNA cases in the present study were classified histologically as FVPTC, none were found to contain the K601E mutation. However, because the hybridization probes were designed as a perfect match to the *BRAF *WT genotype, other mutations covered by the probe would likely lead to a different temperature profile and probable detection of the K601E mutation.

Because the number of samples analyzed in the present study was limited, and the tumor cell content of some of the archival samples was scant, we were unable to determine the full diagnostic utility of LCPCR detection of the *BRAF *activating point mutation on indeterminate thyroid aspirates. Although molecular techniques such as LCPCR may be useful for refining a diagnosis of PTC, the absence of a *BRAF *mutation does not exclude the possibility of a malignant condition. In view of the large number of palpable thyroid nodules that require evaluation by FNA, a search for molecular markers such as *BRAF *may have clinical utility.

The results suggest that detection of BRAF mutation in thyroid aspirates may enhance the accuracy of FNA and refine preoperative diagnosis of PTC.
